# Correlation Between Word Frequency and 17 Items of Hamilton Scale in Major Depressive Disorder

**DOI:** 10.3389/fpsyt.2022.902873

**Published:** 2022-05-03

**Authors:** Jiali Han, Yuan Feng, Nanxi Li, Lei Feng, Le Xiao, Xuequan Zhu, Gang Wang

**Affiliations:** The National Clinical Research Center for Mental Disorders and Beijing Key Laboratory of Mental Disorders, Beijing Anding Hospital and the Advanced Innovation Center for Human Brain Protection, Capital Medical University, Beijing, China

**Keywords:** major depressive disorder, symptom severity, word frequency, temporal lobe, HowNet sentiment dictionary

## Abstract

**Objective:**

To explore the correlation between word frequency and 17 items of the Hamilton Depression Scale (HAMD-17) in assessing the severity of depression in clinical interviews.

**Methods:**

This study included 70 patients with major depressive disorder (MDD) who were hospitalized in the Beijing Anding Hospital. Clinicians interviewed eligible patients, collected general information, disease symptoms, duration, and scored patients with HAMD-17. The words used by the patients during the interview were classified and extracted according to the HowNet sentiment dictionary, including positive evaluation words, positive emotional words, negative evaluation words, negative emotional words. Symptom severity was grouped according to the HAMD-17 score: mild depressive symptoms is 8–17 points, moderate depressive symptoms is 18–24 points and severe depressive symptoms is >24 points. Analysis of Variance (ANOVA) was used to analyze the four categories of words among the groups, and partial correlation analysis was used to analyze the correlation between the four categories of word frequencies based on HowNet sentiment dictionary and the HAMD-17 scale to evaluate the total score. Receiver operating characteristic (ROC) curves were used to determine meaningful cut-off values.

**Results:**

There was a significant difference in negative evaluation words between groups (*p* = 0.032). After controlling for gender, age and years of education, the HAMD-17 total score was correlated with negative evaluation words (*p* = 0.009, *r* = 0.319) and negative emotional words (*p* = 0.027, *r* = 0.272), as the severity of depressive symptoms increased, the number of negative evaluation and negative emotional words in clinical interviews increased. Negative evaluation words distinguished patients with mild and moderate-severe depression. The area under the curve is 0.693 (*p* = 0.006) when the cut-off value is 8.48, the Youden index was 0.41, the sensitivity was 55.2%, and the specificity was 85.4%.

**Conclusion:**

In the clinical interview with MDD patients, the number of word frequencies based on HowNet sentiment dictionary may be beneficial in evaluating the severity of depressive symptoms.

## Introduction

Major depressive disorder (MDD) is a significant public health problem. Studies in recent years have shown that it has a high incidence rate with a lifetime prevalence rate in China at about 3.4% (1), and the global prevalence rate is about 4.4% (2). Likewise, depression has a high disability rate, with 50 million people worldwide affected by depression, accounting for 7.5% of the total global disability rates (2). Depression also has a high disease burden (3), which seriously affects patients' physical health, social functions, and daily living. The current diagnosis process of depression primarily relies on the experience of clinical psychiatrists, professional scale assessments, and subjective descriptions of patients. While these functions are extensively utilized throughout the clinic, these evaluations that rely on subjective analyses and clinical experience may lead to poor consistency between assessments and differences in diagnosis and treatment (4). To thoroughly evaluate the patient, it is integral to determine the severity of depression in patients as part of the clinical diagnosis, especially as these diagnoses are of great significance to patients' treatment plans and assist in identifying life-threatening risks. Therefore, it is urgent to find objective indicators that are easy to collect and apply to assess the symptom severity of depression.

Language is an important way for human beings to transmit information. It is the principal method to transmit ideas and express emotional fluctuations and changes (5, 6). It has a high emotion recognition rate and has the characteristics of easy access, non-invasiveness, and objectivity. It can also reflect people's emotions and cognitive functions. Semantic processing, language, and conceptual categorization depends on temporal lobe. Research showed MDD patients had abnormal results in temporal lobe (7, 8). Temporal lobe injury, especially the amygdala, affects language processing in patients with MDD and lead to negative bias (9). Specifically, patients with depression pay more attention to negative stimuli and less attention to positive ones. Likewise, they are prone to negative interpretations of emotionally ambiguous events or stimuli and often suppress their positive emotional responses (10, 11). Studies have shown that compared with healthy individuals, depressed individuals use more words associated with negative emotions and less associated with positive emotions (12). At present, studies have focused on distinguishing depressive patients from healthy individuals (13, 14) or suicide monitoring (15). Given the lack of studies investigating the recognition of the severity of depressive symptoms using word frequency of depression-related words, this study aimed to evaluate whether the severity of depression symptoms could be measured by word frequency based on the HowNet sentiment dictionary.

## Materials and Methods

### Participants

Subjects recruited for this study were patients with depression hospitalized in the Beijing Anding Hospital from Sep. 2020 to Mar. 2021. The inclusion criteria for this study, in which all 4 items must be met, include (1) patients 18–65 years old with diagnostic criteria of MDD or relapse without psychotic symptoms according to the International Classification of Diseases (ICD-10), (2) 17 items of Hamilton scale (HAMD-17) score >7 points, (3) an education level of primary school or above, and able to understand the content of the scale, and (4) patients that signed the informed consent. The Exclusion criteria were as follows: (1) Patients who met the diagnosis of other mental disorders in ICD-10, (2) Alcohol or drug abuse or dependence within 1 year, (3) currently suffering from oral and throat diseases and other severe physical illness, and (4) mental symptoms were too severe to cooperate with the completion of the research content. This study protocol was approved by the Ethics Committee of the Beijing Anding Hospital. All patients participated in the study and signed informed consent. Finally, 70 patients with MDD were included in this study.

### Measures

We collected general information, including the patient's age, gender, educational level, age at the first onset, frequency of onset, and current course of the disease. HAMD-17 (16) to assess the severity of depressive symptoms in subjects. This scale has good reliability and validity and is widely used to assess the severity of depressive symptoms. The severity of depression was classified according to the total score of HAMD-17: no depressive symptoms are designated as 0–7 points, mild depressive symptoms is 8–17 points, moderate depressive symptoms is 18–24 points and severe depressive symptoms is >24 points. HowNet Sentiment Dictionary (17) is a sentiment dictionary based on the HowNet knowledge database, a robust knowledge database that enables natural language processing systems. This system is a known dictionary for sentiment analysis of Chinese words and is widely used. The dictionary divides vocabulary into four types: 4,534 words of positive evaluation (such as indispensable, sinking fish and geese, beautiful), 1,516 words of positive emotion (such as love, happiness, lingering dreams), and 3,745 words of negative evaluation (such as ugly, excessive, flashy), 1,959 words of negative emotional (such as sad, dubious, dissatisfied).

### Procedure

Clinicians conducted interviews with eligible participants, collected general information, disease symptoms, duration, and participant dialog was recorded using honor 9X mobile. There was no restriction on the time of the interview. Then clinicians who passed the HAMD-17 consistency assessment to judge patients' severity. The audio information was transcribed and manually proofread, and finally, the words used by the patients during the interview were classified into four categories according to the HowNet sentiment dictionary. Finally, divide the four types of words by the interview time to get words per minute.

### Statistical Analysis

SPSS 24.0 software was used for data management and statistical analysis. The normally distributed measurement data were expressed as the mean and standard deviation (mean ± SD), and the non-normally distributed measurement data were expressed as the median and quartile [M (P25, P75)]. Analysis of Variance (ANOVA) was used to analyze the four categories of words between each group, and partial correlation analysis was used to analyze the correlation between word frequencies and HAMD-17 scale total scores. According to the scores with significant differences, the results were divided into Receiver operating characteristic (ROC) curves and were used to determine meaningful cut-off values. *p* values <0.05 were considered to be significant.

## Results

A total of 70 patients participated in this study, including 30 males (42.9%) and 40 females (57.1%), an average of 45.1 (±13.1) years, 7 completed primary school (10.0%), 17 completed junior high school (24.3%), and 45 (64.3%) completed high school and/or above, with 1 case (1.4%) having missing information, 30 cases (42.9%) were designated as first-episode while 40 patients (57.1%) had recurring episodes. The HAMD-17 scale score average was 19.1 (±5.4), of which 29 cases (41.4%) were mild, 27 cases (38.6%) were moderate, and 14 cases (20.0%) were severe. And the average time of the interview is 11.6 (±3.2), the maximum time is 20.2 and the minimal time is 5.1. General information had no differences between groups, as shown in Table 1.

**Table 1:** General information in each group (*n* = 70).

**Item**	**Mild (*n* = 29)**	**Moderate (*n* = 27)**	**Severe (*n* = 14)**	**F/ χ^2^**	***p***
Gender (M/ F)	14/15	12/15	4/10	0.76	0.47
Age	44.5 ± 13.7	46.7 ± 13.6	43.4 ± 11.4	0.33	0.71
Education (years)	12.1 ± 2.8	13.0 ± 3.8	11.2 ± 3.8	1.36	0.26
Age of onset	37.4 ± 13.5	36.3 ± 12.9	39.9 ± 10.4	0.38	0.69
Course (weeks)	16.0 (4.3, 49.5)	26.0 (8, 50)	14.0 (5.8, 22)	1.57	0.47
Number of attacks	2.2 ± 1.5	2.7 ± 2.0	2.1 ± 1.5	0.67	0.52
Interview time (min)	11.8 ± 3.2	11.7 ± 2.9	11.1 ± 3.9	0.21	0.81

Shapiro-Wilk test found that the four types of word frequencies were in line with normal distribution in each group, and the results of ANOVA showed a significant difference in negative evaluation words between groups (*p* = 0.032). Multi-comparison analysis with Least-SignificantDifference (LSD) found a significant difference in negative evaluation words between mild-moderate patients (*p* = 0.022) and mild-severe patients (*p* = 0.036).

**Figure 1:**
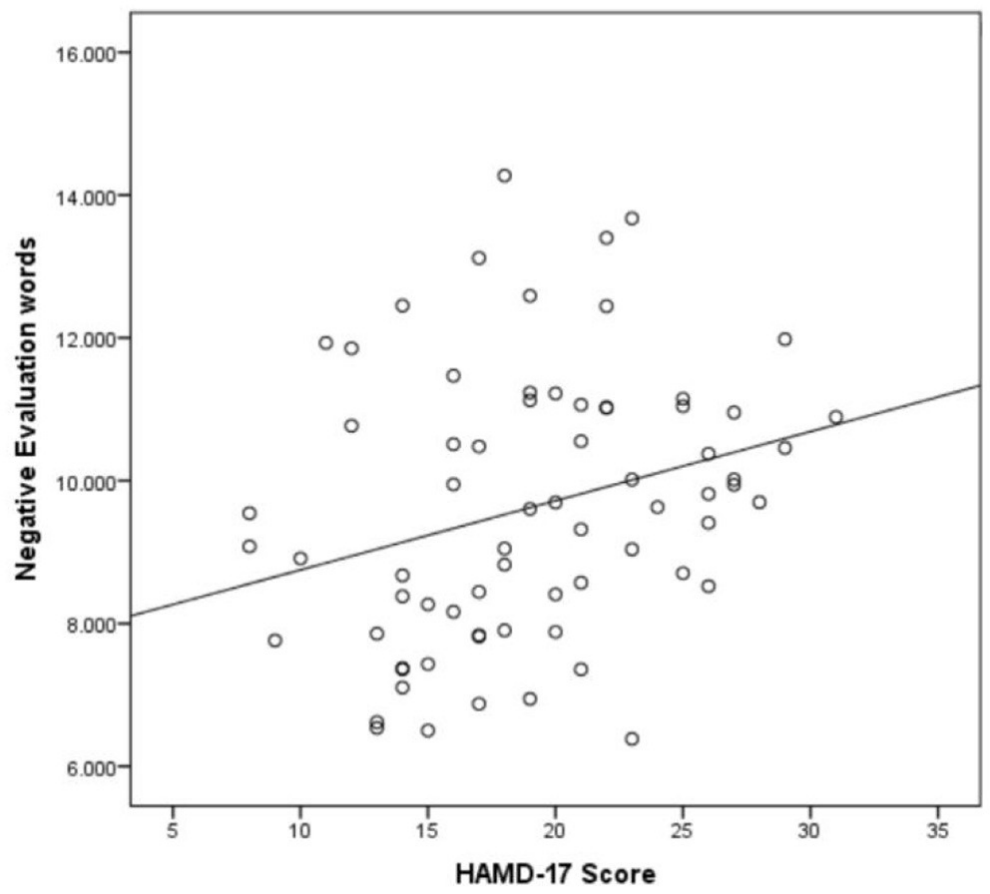
Scatter plot of the association between negative evaluation words per minute and HAMD-17 score (*n* = 70).

**Figure 2:**
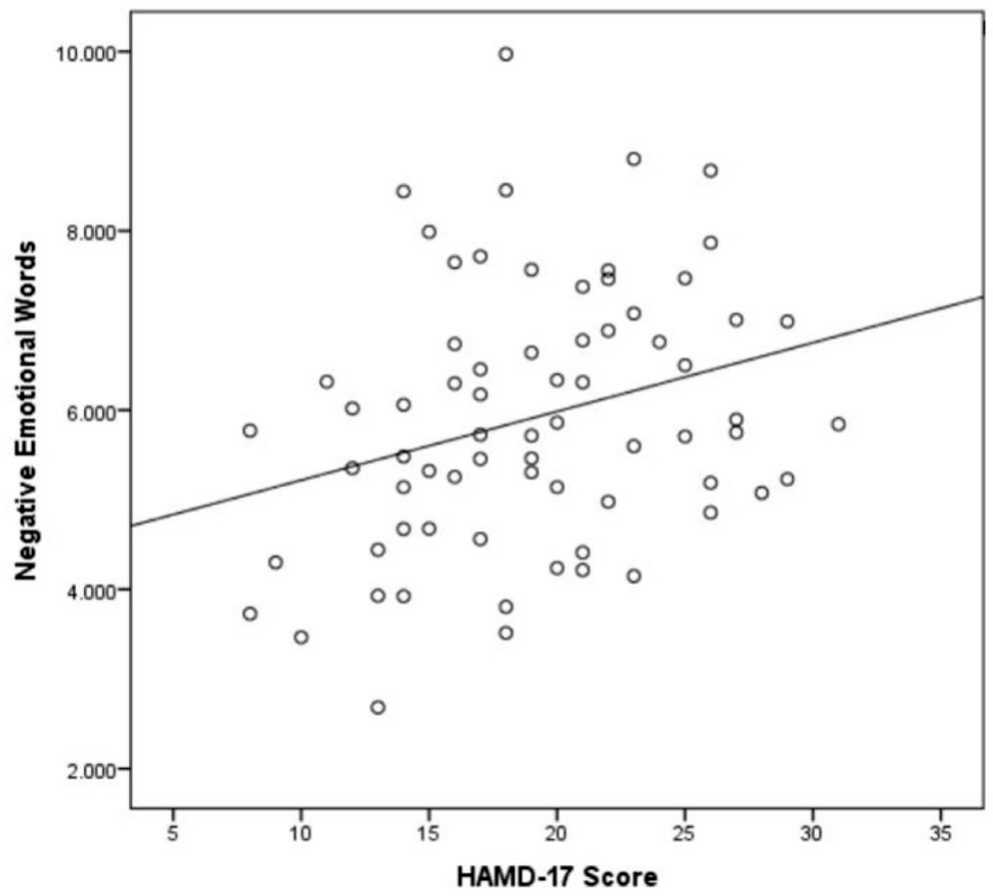
Scatter plot of the association between negative emotional words per minute and HAMD-17 score (*n* = 70).

It was found that there was a correlation between gender, age, education and word frequency. After controlling them, the HAMD-17 total score was correlated with negative evaluation words (*p* = 0.009, *r* = 0.319, Figure 1) and negative emotional words (*p* = 0.027, *r* = 0.272, Figure 2), as shown in Table 2. As the severity of depressive symptoms increased, the number of negative evaluation words and negative emotional words in clinical interviews increased.

**Table 2:** Partial correlation analysis of four types of words.

**Feature**	**Partial correlation**	
	***r***	***p***
Positive evaluation	0.058	0.645
Positive emotion	0.042	0.735
Negative evaluation	0.319^**^	0.009
Negative emotional	0.272^*^	0.027

* *p < 0.05,*

** *p < 0.01*.

**Figure 3:**
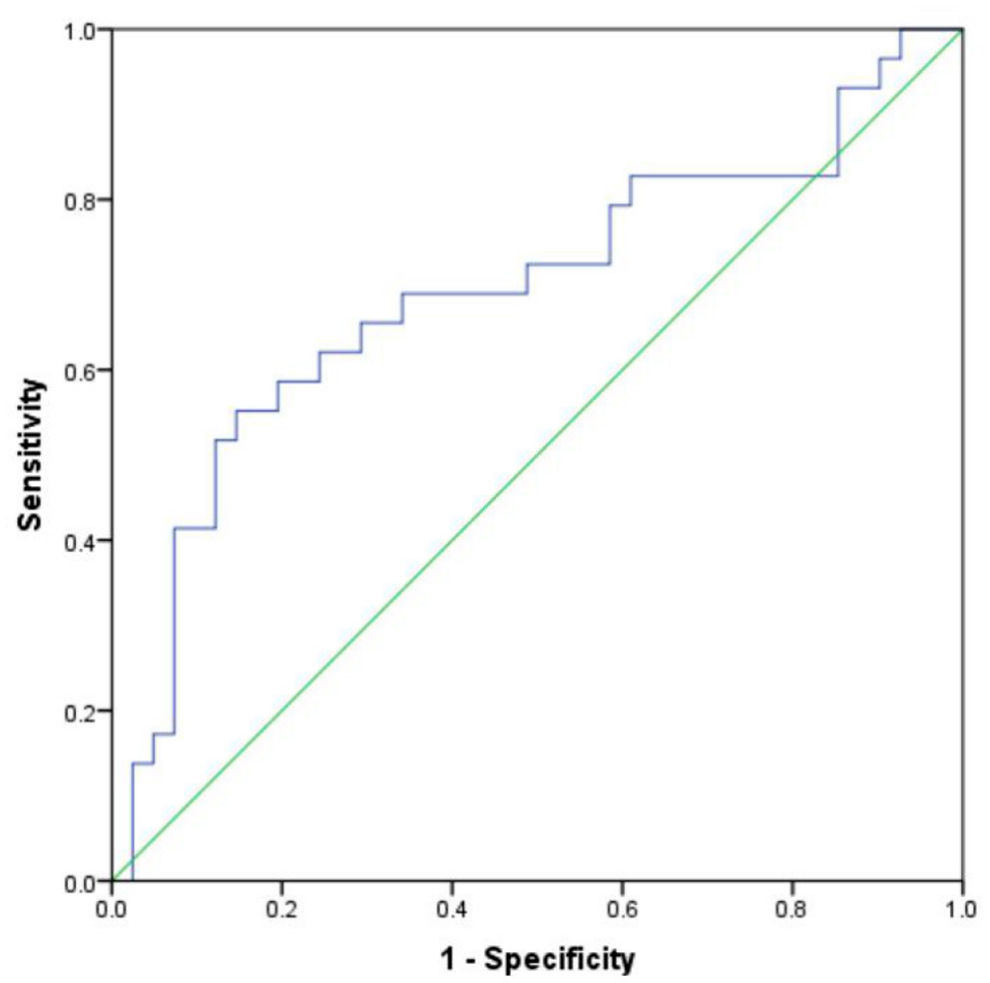
ROC curve analysis of negative evaluation words for differentiating severity of HAMD-17 score.

Based on the predictive value of HowNet sentiment dictionary for the severity of depression, the ROC curve analysis results showed that the area under the curve of negative evaluation words could effectively distinguish patients with mild and moderate-severe was 0.693 (*p* = 0.006). When the cut-off value was 8.48, the Youden index was 0.41, the sensitivity was 55.2%, and the specificity was 85.4% (Figure 3). Therefore, negative evaluation words can effectively distinguish patients with moderate-severe depression.

## Discussion

In this study, we analyzed the word frequency of 70 patients with MDD, and the results of ANOVA showed that the difference in negative evaluation words was significant between groups (*p* = 0.032). Likewise, it was significantly different in patients with mild-moderate patients (*p* = 0.022) and mild-severe (*p* = 0.036) MDD. These findings show that the word frequency feature can distinguish moderate and severe MDD from mild disorders. Notably, the negative evaluation words (*p* = 0.009, *r* = 0.319) and negative emotional words (*p* = 0.027, *r* = 0.272) are positively correlated with the score of HAMD-17. This means that as the severity of depressive symptoms increased, the number of negative evaluation and negative emotional words in clinical interviews increased. Extracting the expressions of negative evaluation words and negative emotion words in patients with MDD has added value in judging the severity of depressive symptoms. It provide some clues to find objective evidence of clinical diagnosis and treatment for other studies in the future. Although patients with MDD have negative emotional expressions, patients often use evaluative words to express their feelings and experiences in clinical interviews. Here, we have identified that the results of negative evaluative words are more significant than negative emotional words.

The results of ROC in this study showed that in the clinical diagnosis and treatment evaluation, the number of negative evaluation words has a significant degree of discrimination for patients with moderate-severe depression. The cut-off value was 8.48, the sensitivity was 55.2%, and the specificity was 85.4%. Since moderate-severe depressive patients need effective treatment and clinical attention, it is beneficial that the high specificity can effectively identify moderate-severe severity from mild.

Previous studies have found that patients with depressive tendencies use more negative emotion words and less (or similar) positive emotion words than others (5, 13, 18–20). But in this study, negative evaluative words were more significant than negative emotional words. This may be due to patients often using evaluative words to express their feelings and experiences in clinical interviews.

Research showed first-episode, drug-naive major depressive disorder and remitted major depressive disorder had differences in temporal gyrus activity. The temporal gyrus may play a critical role in depressive symptomatology (21). This can support that the negative bias of patients with depression is a state index (22). Therefore, it is meaningful to follow patients' word frequency. Past research did not subdivide their depressive symptom severity to explore differences in word usage among depression of different severities. Previous studies have primarily conducted word frequency extraction on people with a tendency to have depression on Weibo or Twitter, and define these subjects as a “patient.” However, physicians have not systematically evaluated the “patients” of those evaluations. Therefore, the lack of necessary clinical details may affect the outcomes of their measures. Yet, in these studies, they are classified as patients with depression only according to words such as “depression” in their text. Therefore, there are some problems in its classification in those studies. To better assess these outcomes in the clinical setting, this study utilized psychiatrists who passed the HAMD-17 consistency assessment to judge patients' severity, allowing for a more reliable diagnosis of disease and severity.

We recognize several limitations of this study. Previous studies conducted data analysis by searching for textual information of patients or non-patients on the Internet. They have a large sample size and cover a wide range of people, while this study was a small sample size for clinical interviews. Therefore, only indicators can be provided, and a machine learning model cannot be established for judging the severity of depression. The proportion of patients with MDD enrolled in this study is 20.0%, which is relatively low, so this may lead to a non-difference between the groups of moderate and severe patients. Although the HowNet sentiment dictionary has been used in a large number of studies, we didn't find literature that measures the reliability and validity of it. This may have an impact on our study. This study only counted the number of the designated four classifications of words. The association between individual word polarity and depressive symptom severity was not assessed. If the polarity of each word and the number of words are analyzed comprehensively, better results may be obtained. Patients who underwent modified electroconvulsive therapy (MECT) within 2 weeks were not excluded. MECT may affect the cognitive level of patients in a short period, affecting the expression and number of their vocabulary, thereby influencing the results of the study. This study is a cross-sectional study with no follow-up survey on patients. To further confirm that the word frequency feature can be used as a variable reflecting the severity of symptoms in patients with MDD, the patients should be followed up to explore whether the number of word frequencies changes when the patients' symptom severity is reduced.

Above all, the problems should be improved in future research to further explore the application value of language information in the symptom assessment of MDD.

## Conclusions

In summary, as the severity of depressive symptoms increased, the number of negative evaluation words and negative emotional words in clinical interviews with MDD patients increased. And the word frequency based on HowNet sentiment dictionary may be beneficial in evaluating the severity of depressive symptoms.

## Data Availability Statement

The original contributions presented in the study are included in the article/supplementary material, further inquiries can be directed to the corresponding author/s.

## Ethics Statement

The studies involving human participants were reviewed and approved by Ethics Committee of Beijing Anding Hospital Affiliated to Capital Medical University. The patients/participants provided their written informed consent to participate in this study.

## Author Contributions

JH, YF, NL, and LF developed the initial idea for the manuscript. JH wrote the main body of the paper and including citations. YF and LX contributed to revision and editing of the manuscript. XZ analyzed the data. GW revised the manuscript. All authors contributed to and have approved the final manuscript.

## Funding

This study was supported by the Beijing Demonstrative Research Ward (Grant No. BCRW202009).

## Conflict of Interest

The authors declare that the research was conducted in the absence of any commercial or financial relationships that could be construed as a potential conflict of interest.

## Publisher's Note

All claims expressed in this article are solely those of the authors and do not necessarily represent those of their affiliated organizations, or those of the publisher, the editors and the reviewers. Any product that may be evaluated in this article, or claim that may be made by its manufacturer, is not guaranteed or endorsed by the publisher.
